# Draft Genome Sequence of Human-Pathogenic *Lactococcus garvieae* LG-ilsanpaik-gs201105 That Caused Acute Acalculous Cholecystitis

**DOI:** 10.1128/genomeA.00464-15

**Published:** 2015-06-04

**Authors:** Ji Hyung Kim, Do-Hyung Kang, Se Chang Park

**Affiliations:** a Biological and Genetic Resources Assessment Division, National Institute of Biological Resources, Incheon, Republic of Korea; b Global Bioresources Research Center, Korea Institute of Ocean Science & Technology, Ansan, Republic of Korea; c Laboratory of Aquatic Biomedicine, College of Veterinary Medicine and Research Institute for Veterinary Science, Seoul National University, Seoul, Republic of Korea

## Abstract

*Lactococcus garvieae*, which is generally known as a marine and freshwater fish pathogen, is now considered to be an emerging zoonotic pathogen in both human and veterinary medicine. In recent years, we have reported the infection of *L. garvieae* LG-ilsanpaik-gs201105 in the gallbladder of an old fisherman. In this study, we present the draft genome sequence of *L. garvieae* LG-ilsanpaik-gs201105, with a total genome size of 1,960,261 bp in 53 contigs and a 38.1% average G+C content. Interestingly, the capsule gene cluster, which was known as one of the crucial virulence factors in *L. garvieae*, was not detected in our isolate. This is the first genome sequence of human-pathogenic *L. garvieae*, which caused acute acalculous cholecystitis.

## GENOME ANNOUNCEMENT

*Lactococcus garvieae* is a Gram-positive bacterium that causes septicemia in various marine and freshwater fish (1) and subclinical mastitis in ruminants (2). In recent years, this bacterium has been considered to be an emerging zoonotic pathogen due to an increase in fatal human infections (3). Previously, we reported the first case of acute acalculous cholecystitis caused by *L. garvieae* in a 69-year-old fisherman (4). The isolate, obtained from the fisherman's gallbladder, was identified as *L. garvieae* and finally designated *L. garvieae* LG-ilsanpaik-gs201105.

In this study, we present the genome sequence of *L. garvieae* LG-ilsanpaik-gs201105. The genomic DNA was isolated using the DNeasy blood and tissue kit (Qiagen), and sequencing was performed using the Roche/454 pyrosequencing method on the Genome Sequencer FLX system with Titanium chemistry at Macrogen, Inc. in South Korea (40× coverage). Putative open reading frames (ORFs) were predicted using the Web-based NCBI Glimmer version 3.02 (5) and GeneMark.hmm (6), and translated ORFs were then compared to known protein sequences using BLAST. Gene ontology (GO) databases were additionally used to functionally classify the ORFs. Bacterial tRNAs and rRNAs were analyzed using tRNAscan-SE 1.21 (7) and RNAmmer 1.2 (8), respectively.

The sequenced data consisted of a total of 154,335,000 bp and 232,924 reads, with an average read length of 662.6 bp. Furthermore, the data include 225,320 assembled reads and 3,894 partially assembled reads. Using GS *de novo* assembler (version 2.6) software, the reads were assembled into 97 contigs, and a total of 53 contigs >500 bp were finally recovered. The assembled genome of *L. garvieae* LG-ilsanpaik-gs201105 was 1,960,261 bp, and its average G+C content was estimated to be 38.1%. A total of 1,899 ORFs were detected in the bacterial genome, and its GO analysis results revealed that 33.7%, 37.9%, and 6.3% of the sequences included genes related to biological processes, molecular functions, and cellular components, respectively. In the GO category of biological processes, metabolic processes was the predominant subcategory, representing 39.5% of the genes. In the cellular component category, 48.6% of the genes were annotated as unknown, but 23.5% and 12.6% of the genes were associated with cell parts and membrane, respectively. Based on their molecular function, 40% of the genes were identified as being associated with catalytic activity. Additionally, 46 tRNAs and 4 rRNAs were analyzed from the bacterial genome. Interestingly, the closest relative of our isolate was *L. garvieae* Lg2 (GenBank accession no. NC_017490), which was reported as a virulent strain; however, the capsule gene cluster, which was known as one of the crucial virulence factors in *L. garvieae* (9), was not detected in *L. garvieae* LG-ilsanpaik-gs201105. This is the first genome sequence of a human-pathogenic *L. garvieae* strain that caused acute acalculous cholecystitis. 

### Nucleotide sequence accession numbers.

The data from this whole-genome shotgun project have been deposited at DDBJ/EMBL/GenBank under the accession no. JPUJ00000000. The bacterium has been deposited in the Korean Culture Center of Microorganisms (KCCM) with deposit no. KCCM 90122.
